# Thread shape, cortical bone thickness, and magnitude and distribution of stress caused by the loading of orthodontic miniscrews: finite element analysis

**DOI:** 10.1038/s41598-022-16662-w

**Published:** 2022-07-20

**Authors:** Takuma Sakamaki, Keiichiro Watanabe, Akihiko Iwasa, Toru Deguchi, Shinya Horiuchi, Eiji Tanaka

**Affiliations:** 1 Department of Orthodontics and Dentofacial Orthopedics, Tokushima University Graduate School of Oral Sciences, Tokushima, Japan; 2 Department of Orthodontics and Dentofacial Orthopedics, Tokushima University Graduate School of Biomedical Sciences, 3-18-15 Kuramoto-cho, Tokushima, 770-8504 Japan; 3 Division of Orthodontics, College of Dentistry, The Ohio State University, Columbus, OH USA

**Keywords:** Dental materials, Orthodontics

## Abstract

Cortical bone thickness is assumed to be a major factor regulating miniscrew stability. We investigated stress distribution in two miniscrews with different thread shapes (type A and B) and in cortical bone of three different thicknesses using three-dimensional (3D) finite element (FE) models. More specifically, 3D FE models of two different miniscrews were created and placed obliquely or vertically into a cylindrical bone model representing different cortical bone thicknesses. When force was applied to the miniscrew, the stress distribution on the screw surface and in the peri-implant bone was assessed using FE methodology. Miniscrew safety was evaluated using a modified Soderberg safety factor. Screw head displacement increased with a decrease in cortical bone thickness, irrespective of screw type. The smallest minimum principal stresses on the screw surfaces remained constant in type A miniscrews on changes in cortical bone thickness. Minimum principal stresses also appeared on the cortical bone surface. Lower absolute values of minimum principal stresses were seen in type A miniscrews when placed vertically and with upward traction in obliquely placed type B miniscrews. Both miniscrews had acceptable safety factor values. Taken together, orthodontists should select and use the suitable miniscrew for each patient in consideration of bone properties.

## Introduction

In clinical orthodontics, miniscrews are implanted directly into the bone and offer absolute anchorage for different types of tooth movement^1,2^. They also provide biocompatibility, reduced discomfort, less invasiveness, and few limitations in placement as compared with miniplates^3,4^. Notwithstanding their small diameter and short length, miniscrews are an efficient modality available within modern orthodontic treatment^1,2,5^. For these reasons, miniscrews are widely accepted by both orthodontists and patients^6,7^.

On the other hand, the clinical use of miniscrews carries some risks and concerns. Screw failure might be one of the most undesirable adverse effects in the clinical use of miniscrews^8^. Even adequately placed miniscrews may fail. The success rate of miniscrews has been reported to be approximately 80%^8^. When considering the higher success rate of dental implants (which is reported to range from 96 to 99%), the failure rate of orthodontic miniscrews is comparably higher^9^. Moreover, screw failure is likely to occur early after placement. Thus, the primary stability is the most common parameter associated with screw failure and its enhancement is an urgent issue for clinical practitioners.

The primary stability of orthodontic miniscrews is associated with many factors^9–15^. For example, studies report that the primary stability of orthodontic miniscrews is closely related to the properties of the cortical bone, the screw design, inflammation in the peri-implant tissue, and the applied force^16–18^. Cortical bone fragility is generally assumed to be a major factor regulating miniscrew stability^19,20^, and cortical bone thickness is an essential factor in the successful placement of miniscrews as thinner cortical bone has insufficient primary stability^21,22^. Low bone density is another common cause of screw failure^23^.

Various clinical features can be simulated using three-dimensional (3-D) finite element (FE) models evaluating the distribution and magnitude of stress induced in the miniscrew and peri-implant bone. A high concentration of stress overloads the surrounding bone and stimulates bone microfracture in the region^24^. The distribution and magnitude of stress in the bone can be used to investigate the effectiveness of miniscrews and may be predictive of the risk of screw failure^25–27^. Ghorbanyjavadpour et al.^28^ investigated the stress produced during miniscrew insertion into bone using a 3-D FE model and suggested that most stress and strain was well tolerated by the cortical bone, but not by the cancellous bone. However, little information is available about the effects of cortical bone thickness on stress distribution and screw stability.

Previously, we evaluated the effects of cortical bone thickness on the mechanical stability of miniscrews in an in vitro study and found that the mechanical stability of miniscrews increased when the screws were inserted into a thicker articular bone block^29,30^. However, mechanical properties were found to differ meaningfully between miniscrews with different thread shapes, irrespective of the cortical bone thickness ^30^. Thus, this research aimed to evaluate the stress distribution in two types of miniscrews with different thread shapes as well as in surrounding bone with three different thicknesses of cortical bone when miniscrews were inserted vertically or obliquely; these parameters were evaluated using 3-D FE models.

## Methods

In the present study, we adopted two different titanium miniscrews with the same screw length and diameter: type A (AbsoAnchor, SH1615-07; Dentos Ltd., Daegu, South Korea) and type B (B-max Screw, Type-TK; BIODENT Co., Tokyo, Japan) miniscrews. Both miniscrews were made of titanium alloy (6AL-4V ELI) and had a screw length of 7.0 mm and a screw diameter of 1.6 mm. The yield stress of the titanium alloy was reported to be 755 MPa, and the fatigue endurance limit was 412.5 MPa^31^.

The type A miniscrew had a shaft length of 5.5 mm (the pitch and depth of a traditional thread with a buttress reverse thread shape), with a taper of 1.30°. Micro-computed tomographic (CT) images were acquired with a cubic voxel of 5 μm^3^, and serial images of 845 consecutive slices were used to reconstruct the 3-D surface model using a CT modeler (Toshiba IT & Control Systems Co., Tokyo, Japan); 3-D finite elemental models of the type A miniscrew were constructed from the segmental images.

The type B miniscrew had a shaft length of 5.2 mm, a pitch of 0.5 mm, a depth of 0.2 mm, and a taper of 2.0°. The screw thread was characterized according to a 35° proximal half angle and a 10° distal half angle; 3-D FE models of the type B miniscrews were created using computer-aided design data provided by BIODENT Co., Ltd.

**Figure 1:**
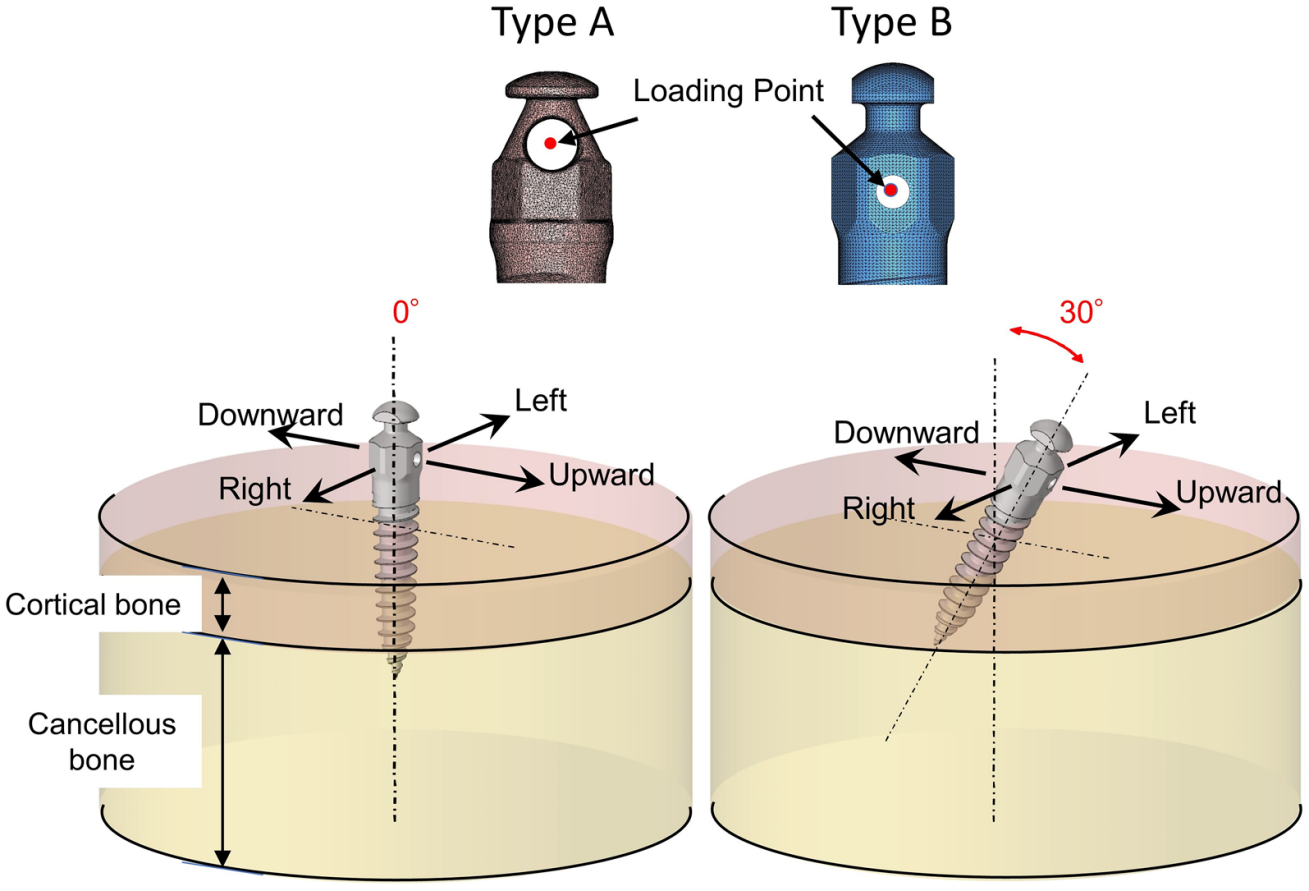
Schematic illustration of 3D finite element models for the evaluated miniscrews. The modeled cortical bone had a thickness of 1.5 mm, 2.0 mm, or 3.0 mm. The orthodontic miniscrews were placed at a direction of 0° or 30°.

The peri-implant bone model had a cylindrical shape. It consisted of cortical bone and cancellous bone layers. Three peri-implant bone models were created to represent cortical bone thicknesses of 1.5 mm, 2.0 mm, and 3.0 mm, and cancellous bone thicknesses of 5.5 mm, 5.0 mm, and 4.0 mm, respectively (Fig. 1). The miniscrew models were placed to a depth of 5.5 mm. These miniscrew models were constructed to represent screw placement orientations of 0° (vertical direction) and 30° (oblique direction) against the cortical bone surface (Fig. 1). Twelve models were meshed with delta cone tetrahedrons representing the cortical and cancellous bones using HyperWorks software (Altair Engineering, Troy, MI, USA).

All solid elements were isotropic, homogeneous, and linear elastic. Table 1 shows the mechanical properties of the cortical and cancellous bone and of the miniscrews^31–34^. As the boundary condition, the bottom and the peripheral edge of the bone model were constrained for all degrees of freedom in order to avoid any movements of the entire model. The interfaces between the miniscrew and cortical and cancellous bones were established with nonfrictional contact elements. Due to these elements, the two contacting surfaces were able to move independently and without penetration.

**Table 1:** Mechanical properties of three different modeled materials.

	Young’s modulus E (GPa)	Poisson ratiov	Yield strength (MPa)
Miniscrew (6AL-4V ELI)	114.0	0.34	755
Cortical bone	14.7	0.30	150
Cancellous bone	1.5	0.30	15

For the loading condition, orthodontic force was applied at the center of the screw head hole in four different directions in each model (Fig. 1). The amount of traction force was 2 N, which is similar to the force commonly applied to miniscrews during orthodontic tooth movements^35,36^. The direction of force application was defined with respect to the cortical bone surface. The principal stresses were calculated for all nodes of the screw and bone. The distribution of stress in the surrounding bone and on the screw surface as well as the displacement of the point of the screw head where the load had been applied were evaluated using the FE analysis program Nastran (Autodesk Nastran version 2018, Autodesk, San Rafael, CA, USA).

Localized stress concentration causes fatigue failure of titanium alloy products^37^. Failure can occur under tension and/or compression conditions, particularly when tension and/or compression are applied in less mechanically demanding regions^38,39^. Thus, to evaluate the fatigue life of the miniscrews under variable loading directions, the local Soderberg safety factors (*S*_*f*_) were calculated at the point wherein the greatest maximum principal stress was induced according to the following formula, considering both the first and third principal stresses (δ_*I*_ and δ_*III*_) indicating the maximum principal tensile and compressive stresses, respectively^40^:

$$S_{f} \, = \,{1}/(\delta_{a} /\delta_{f} \, + \,\delta_{m} /\delta_{y} )$$

where $\delta_{a} = \left| {\delta_{I} {-}\delta_{III} } \right|/{2}$ represents the stress amplitude and δ_*m*_ = (δ_*I*_ + δ_*III*_)/2 represents the mean stress. Moreover, δ_*f*_ and δ_*y*_ represent the fatigue endurance limit and yield stress for the titanium alloy (6AL-4V ELI), respectively.

## Results

### Screw head displacement

**Figure 2:**
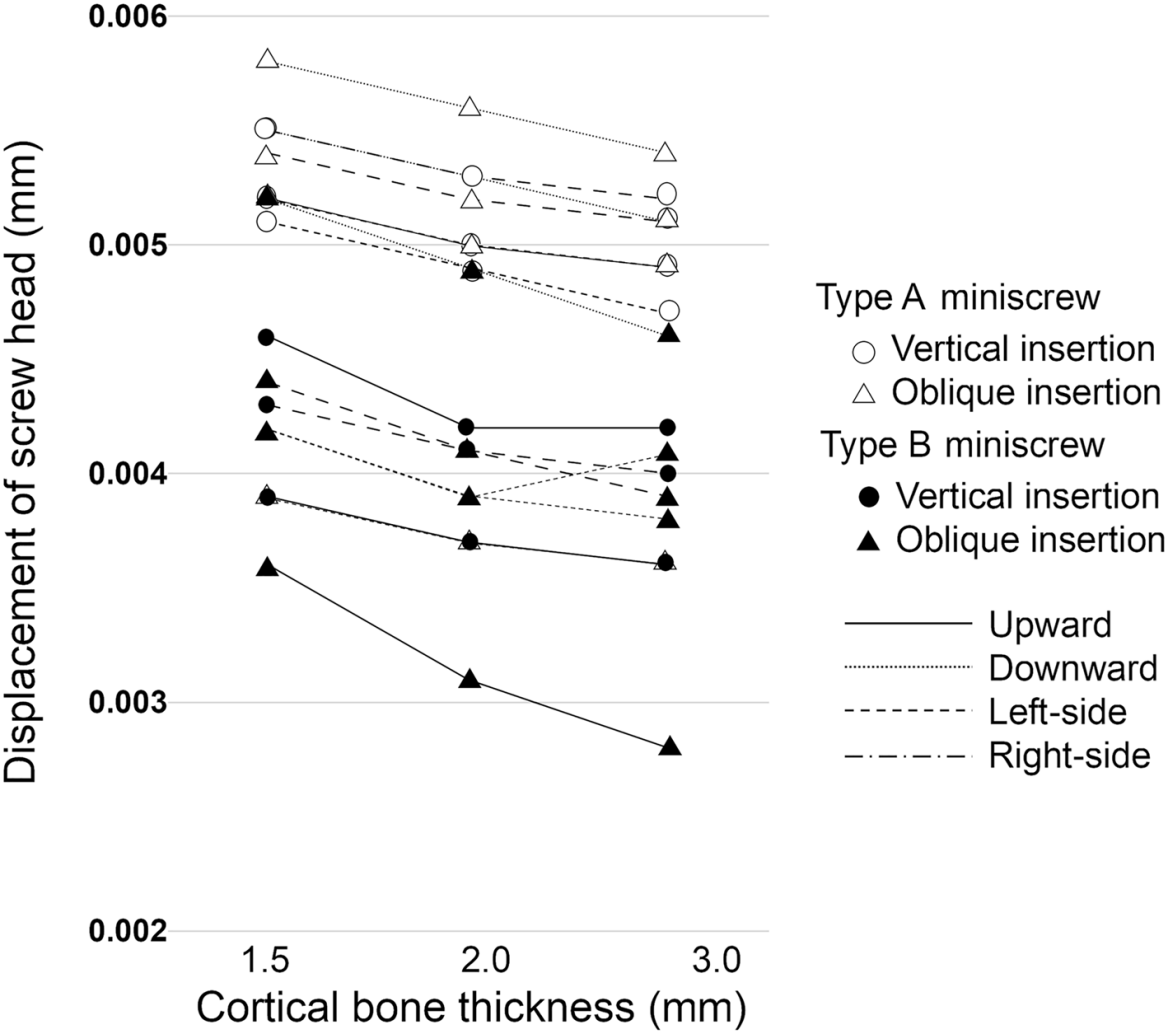
Displacement of the loading points. Type A miniscrew: ○ vertical insertion, △ oblique insertion. Type B miniscrew: ● vertical insertion, ▲ oblique insertion.  Upward  Downward  Left-side Right-side.

Regardless of the insertion direction and screw type, we found that screw head displacement increased as the cortical bone thickness decreased (Fig. 2). Moreover, type A miniscrews showed larger displacement than did type B miniscrews under the corresponding condition.

When miniscrews were placed vertically, the screw head was displaced similarly regardless of the loading direction. When the cortical bone was 1.5 mm thick, the screw head displacement was 0.0053 mm and 0.0042 mm on average in type A and B miniscrews, respectively. On the other hand, when the miniscrew was placed obliquely, the screw head displacement was smallest with upward traction, irrespective of the screw type and cortical bone thickness. When loading was applied upward, the displacement of the screw head was 0.0036–0.0039 mm in type A miniscrews. In type B miniscrews, the displacement was 0.0028–0.0036 mm.

### Distribution of principal stress on the miniscrew surface

**Figure 3:**
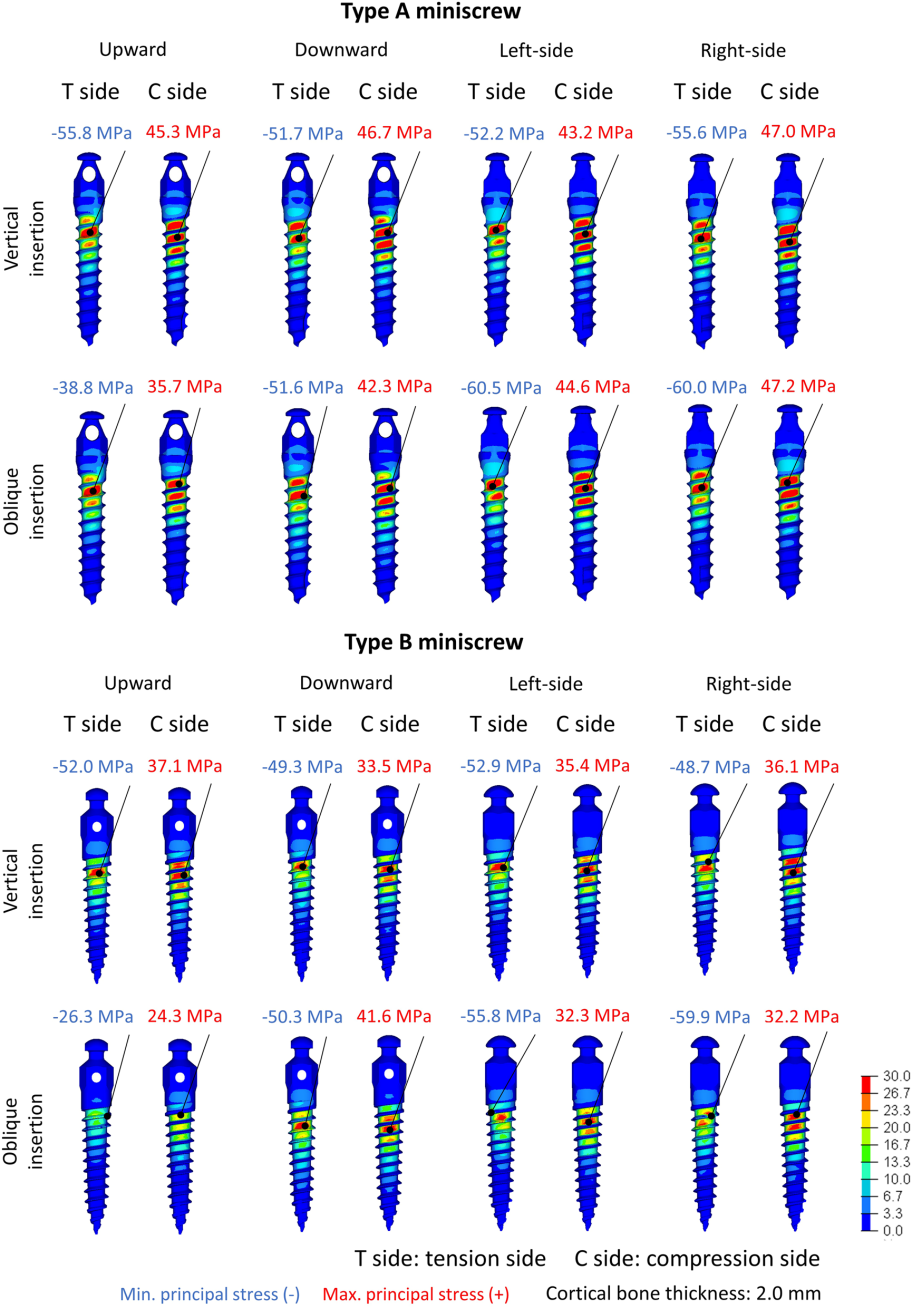
Distribution of maximum and minimum principal stresses on the miniscrew surface on the loaded side when the miniscrew was placed vertically or obliquely in a model of 2.0 mm thick cortical bone.

Figure 3 and Table 2 show the representative figurations of the maximum and minimum principal stresses and the values of greatest maximum and smallest minimum principal stresses, respectively. When the screws were inserted vertically, both the greatest maximum and smallest minimum principal stresses were located at the first or second thread. Moreover, the maximum and minimum principal stresses extended throughout the fifth or sixth thread regardless of the screw type and loading direction. In addition, in type A miniscrews, the absolute values of smallest minimum principal stresses remained virtually constant (ranging from 38.4 to 60.5 MPa) regardless of the thickness of the cortical bone. In type B miniscrews, these values ranged from 26.3 to 59.9 MPa and were lower than those observed for type A miniscrews.

**Table 2:** Greatest maximum and smallest minimum principal stresses (MPa) on the miniscrew surface when the miniscrew was placed vertically or obliquely in each cortical bone thickness.

Cortical bone thickness	Insertion direction		Loading direction			
			Upward	Downward	Left-side	Right-side
**Type A miniscrew**						
1.5	Vertical	Maximum	46.2	47.7	44.3	46.1
		Minimum	− 54.4	− 52.4	− 50.4	− 58.9
	Oblique	Maximum	35.7	41.1	45.7	48.2
		Minimum	− 38.4	− 51.3	− 58.7	− 57.4
2.0	Vertical	Maximum	45.3	46.7	43.2	47.0
		Minimum	− 55.8	− 51.7	− 52.2	− 55.6
	Oblique	Maximum	35.7	42.3	44.6	47.2
		Minimum	− 38.8	− 51.6	− 60.5	− 60.0
3.0	Vertical	Maximum	46.3	47.0	44.5	47.0
		Minimum	− 55.4	− 53.1	− 54.1	− 58.7
	Oblique	Maximum	35.4	40.1	44.3	45.4
		Minimum	− 39.0	− 51.5	− 53.8	− 56.3
**Type B miniscrew**						
1.5	Vertical	Maximum	35.1	35.7	35.2	33.9
		Minimum	− 56.7	− 43.0	− 48.7	− 48.6
	Oblique	Maximum	25.8	39.0	34.2	35.5
		Minimum	− 38.2	− 54.7	− 49.7	− 45.3
2.0	Vertical	Maximum	37.1	33.5	35.4	36.1
		Minimum	− 52.0	− 49.3	− 52.9	− 48.7
	Oblique	Maximum	24.3	41.6	32.3	32.2
		Minimum	− 26.3	− 50.3	− 55.8	− 59.9
3.0	Vertical	Maximum	34.7	31.2	33.9	34.3
		Minimum	− 45.0	− 45.1	− 54.8	− 45.8
	Oblique	Maximum	24.6	33.2	31.9	31.4
		Minimum	− 28.6	− 51.6	− 45.4	− 48.9

When miniscrews were implanted obliquely, the maximum and minimum principal stresses also spread widely, and the smallest minimum principal stress concentrated on the first or second thread. The absolute value of smallest minimum principal stress was smallest with upward traction, irrespective of the cortical bone thickness and the screw type. The absolute value of smallest minimum principal stress was smaller for type B miniscrews (mean, 31.0 MPa) than for type A miniscrews (mean, 38.7 MPa). Moreover, for type B miniscrews, when traction force was applied upward, the values representing the absolute value of smallest minimum principal stresses decreased as the cortical bone became thicker. When loading was applied in any other direction, the values representing the greatest maximum and smallest minimum principal stresses were almost identical irrespective of the cortical bone thickness and the screw type.

### Principal stress distribution in the cortical bone

**Figure 4:**
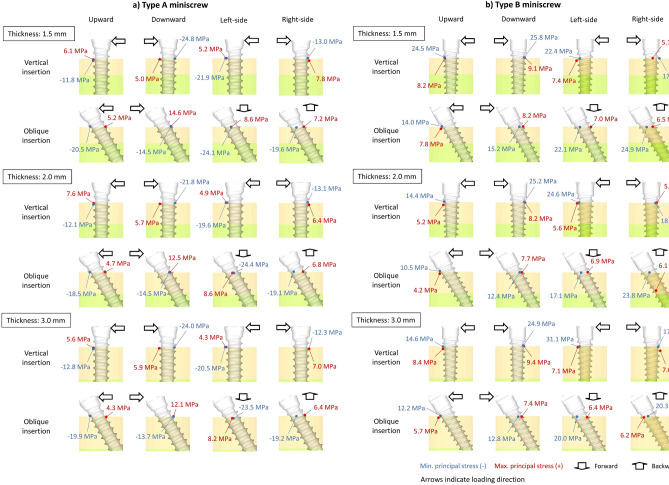
Schema of greatest maximum and smallest minimum principal stresses on the surface of cortical bone when the miniscrew was placed vertically or obliquely in each cortical bone thickness. (**A**) Type A miniscrew, (**B**) Type B miniscrew. Arrows indicate the loading direction.

The greatest maximum principal stress was concentrated on the surface of the cortical bone corresponding to the first or second screw thread (Fig. 4 and Table 3). When the miniscrew was placed vertically, the greatest maximum principal stresses showed relatively small changes compared to the smallest minimum principal stresses (which ranged from 4.3 to 8.2 MPa) irrespective of the screw type, force direction, and cortical bone thickness.

**Table 3:** Greatest maximum and smallest minimum principal stresses (MPa) on the surface of the modeled cortical bone when the miniscrew was placed vertically or obliquely in each cortical bone thickness.

Cortical bone thickness (mm)	Insertion direction		Loading direction			
			Upward	Downward	Left-side	Right-side
**Type A miniscrew**						
1.5	Vertical	Maximum	6.1	5.0	5.2	7.8
		Minimum	− 11.8	− 24.8	− 21.9	− 13.0
	Oblique	Maximum	5.2	14.6	8.6	7.2
		Minimum	− 20.5	− 14.5	− 24.1	− 19.6
2.0	Vertical	Maximum	7.6	5.7	4.9	6.4
		Minimum	− 12.1	− 21.8	− 19.6	− 13.1
	Oblique	Maximum	4.7	12.5	8.6	6.8
		Minimum	− 18.5	− 14.5	− 24.4	− 19.1
3.0	Vertical	Maximum	5.6	5.9	4.3	7.0
		Minimum	− 12.8	− 24.0	− 20.5	− 12.3
	Oblique	Maximum	4.3	12.1	8.2	6.4
		Minimum	− 19.9	− 13.7	− 25.2	− 19.2
**Type B miniscrew**						
1.5	Vertical	Maximum	8.2	9.2	7.4	5.1
		Minimum	− 24.5	− 25.8	− 22.4	− 17.0
	Oblique	Maximum	7.8	8.2	7.0	6.5
		Minimum	− 14.0	− 15.2	− 22.1	− 24.9
2.0	Vertical	Maximum	5.2	8.2	5.6	5.0
		Minimum	− 14.4	− 25.2	− 24.6	− 18.5
	Oblique	Maximum	4.2	7.7	6.9	6.1
		Minimum	− 10.5	− 12.4	− 17.1	− 23.8
3.0	Vertical	Maximum	8.4	9.4	7.1	7.0
		Minimum	− 14.6	− 24.9	− 31.1	− 17.5
	Oblique	Maximum	5.7	7.4	6.4	6.2
		Minimum	− 12.2	− 12.8	− 20.0	− 20.3

The smallest minimum principal stresses also appeared on the cortical bone surface corresponding to the first screw thread (Fig. 4). In both miniscrew types, the smallest minimum principal stress varied largely, ranging from − 25.8 to − 10.5 MPa. When type A miniscrews were placed vertically, the absolute values of the minimum principal stresses were greater with upward and right-side traction (as compared to those with downward and left-side traction) regardless of the cortical bone thickness. As the cortical bone became thicker, the absolute values of the minimum principal stresses did not increase given any direction of force application. When type B miniscrews were placed vertically, the absolute values of the smallest minimum principal stresses were larger than those of type A miniscrews. When type A miniscrews were placed obliquely, the absolute values of the minimum principal stress were smaller with downward traction compared to those with traction in any other direction. This trend was independent of the cortical bone thickness (Fig. 4). When comparing the two different miniscrews under vertical placement conditions, the absolute values for minimum principal stress were lower for type A miniscrews than for type B miniscrews in all force directions. When the miniscrews were placed obliquely, type B miniscrews exhibited smaller absolute values for minimum principal stress with upward traction.

### Miniscrew safety using Soderberg safety factors

**Figure 5:**
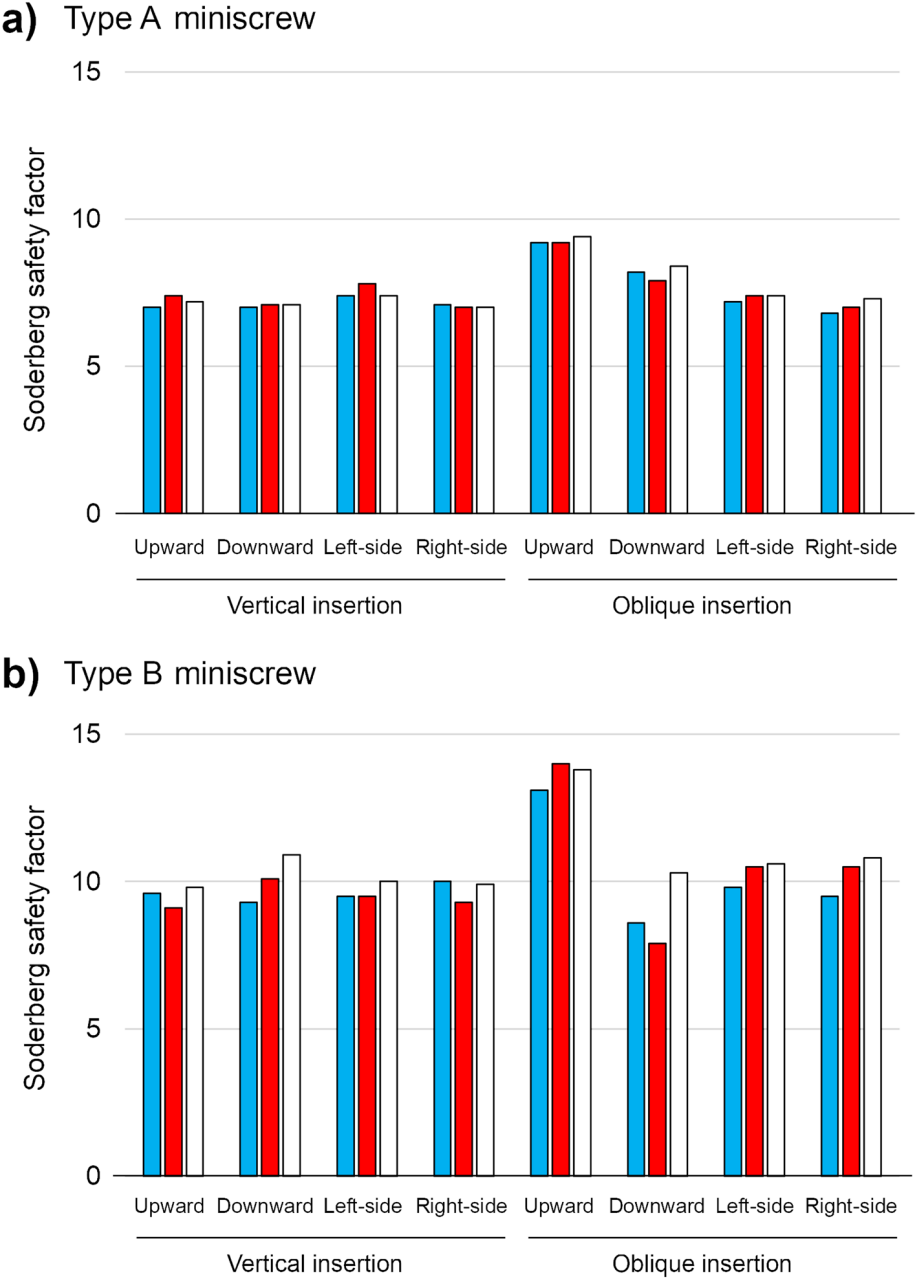
Soderberg safety factor values for different types of miniscrews when the miniscrew (placed vertically or obliquely) was loaded in various directions. (**A**) Type A miniscrew, (**B**) Type B miniscrew.  1.5 mm,  2.0 mm,  3.0 mm.

Soderberg safety factors were computed at the point where the greatest maximum principal stress was induced. Regardless of screw type and cortical bone thickness, these values remained almost constant when the miniscrews were implanted vertically (Fig. 5). When the screws were implanted obliquely, the Soderberg safety factor values were dependent on the direction of force application.

For type A miniscrews, the minimum safety factor was 6.8 around the second screw thread when the miniscrew was implanted obliquely into cortical bone with a 1.5 mm thickness and with right-side traction, while the maximum safety factor was 9.4 around the second screw thread when the screw was implanted obliquely into cortical bone with a 3.0 mm thickness and with upward traction. When the miniscrews were placed obliquely, the greatest Soderberg safety factor value was detected with upward traction regardless of the cortical bone thickness.

For type B miniscrews, the minimum safety factor was 7.9 around the third thread when the screw was inserted obliquely into cortical bone with a 2.0 mm thickness and with downward traction, while the largest safety factor was 14.0 around the first thread when the screw was placed obliquely into cortical bone with a 2.0 mm thickness and with upward traction. The Soderberg safety factor values were larger than those of type A miniscrews under the corresponding condition. When the miniscrews were placed obliquely, the maximum and minimum safety factor values were found upon upward and downward traction, respectively.

## Discussion

In the clinic, orthodontists often face situations that require tooth intrusion, such as incisor intrusion in cases of deep bite with gummy smile, molar intrusion in cases of anterior open bite, and correction of the canted occlusal plane, among other clinical situations. To treat these malocclusions, obliquely inserted miniscrews with upward traction can be considered effective under orthodontic loading^41^. The present study assessed the magnitude and distribution of stress induced in two types of miniscrews with different thread shapes and in peri-implant bone with three different thicknesses of cortical bone using 3D FE models. We found lower absolute values of minimum principal stress when type A miniscrews were placed vertically, when type B miniscrews were placed obliquely, and under upward traction. Both miniscrews had acceptable safety factor values.

To date, various types of miniscrews have been developed and distributed worldwide; their length ranges from 5 to 12 mm, and their diameter ranges from 1.3 to 2.0 mm^8^. Only type B miniscrew used in this study has a different thread shape. The present study evaluated the stress distribution in the two miniscrews with different thread shape and in cortical bone using 3D FE models, and our results indicated the effect of thread shape on the safety and initial stability of the miniscrew. However, since there may be another miniscrew that may show better performance than the two miniscrews used in this study, further investigations using much more types of miniscrews are required to determine the optimal design of miniscrew for the advantage of safety and secure stability.

Since miniscrews are commonly used for approximately 2 years during orthodontic treatment, fatigue behavior must be considered during the design phase of these structures in regard to adequate estimation of safety factors and lifetime^42^. In the present study, we adopted the Soderberg approach to compute safety factors under fatigue loads^43,44^. A safety factor of 2.0 indicates that a miniscrew could withstand a stress level two times greater than that which it would realistically meet in its lifetime^44^. In the present study, although type A miniscrews revealed slightly lower design safety factor values, the safety factor values were acceptable for both types of miniscrews. This indicates that both types of miniscrews were appropriately designed to prevent screw fracture and to improve screw performance during orthodontic treatment. Not all miniscrew designs can be utilized in all patients due to individual differences in cortical bone thickness and bone density. Therefore, different types of miniscrews are commercially available, such as cylindrical and tapered miniscrews and stainless-steel and titanium alloy miniscrews. In addition, in this study, regardless of the screw type, we found that Soderberg safety factor values were greatest with upward traction when miniscrews were implanted obliquely. Therefore, it is advisable to adopt oblique insertion of the miniscrew in order to prevent its fracture.

The primary stability of orthodontic miniscrews is closely related to the thickness of the surrounding cortical bone^16,30,39^. Han et al.^30^ indicated that the mechanical stability of miniscrews increased when miniscrews were installed in artificial bone blocks of the same density but greater thickness. Our results showed that the displacement of the screw head was greater when the miniscrews were placed in a bone model with a thinner cortical bone layer than in bone models with a thicker cortical bone layer, irrespective of the miniscrew type. Moreover, the type A miniscrew showed greater displacement of the screw head than did the type B miniscrew. This is due to differences in the height from the cortical bone surface to the loading point, which causes differences in the center of gravity of the miniscrew. Regarding the stress distribution on the surface of the miniscrew, the smallest minimum principal stress remained almost constant when the miniscrew was placed vertically regardless of cortical bone thickness, indicating that the minimum principal stress induced on the surface of the screw thread is insulated from the effect of cortical bone thickness. Moreover, when type A miniscrews were placed obliquely, the values of the smallest minimum principal stresses on the screw surface remained steady irrespective of the cortical bone thickness, although the absolute value of the smallest minimum principal stress was smallest with upward traction. In type B miniscrews, the absolute values of the smallest minimum principal stresses were 5–30% smaller than those in type A miniscrews. Moreover, the absolute value of the smallest minimum principal stress was the smallest when type B miniscrews were placed obliquely and with upward traction.

In addition, as the cortical bone became thicker, the maximum and absolute value of minimum principal stresses decreased markedly. This may be because the active contact area between the miniscrew and the cortical bone increased when the miniscrew was implanted obliquely and with upward traction. This increase in the contact area between the miniscrew and cortical bone results in a widespread distribution of the applied force and reduces the stress concentration. Pan et al.^45^ reported that the cortical bone thickness at an angle of 30° against the long axis of the tooth was approximately 1.5 times larger than that at an angle of 90°. Previously, we evaluated the influence of placement angles ranging from 0° to 45° on the initial stability of type A miniscrew and demonstrated that the stresses in the surrounding cortical bone increased with the angle of insertion^41^. This indicates that, as the insertion angle increases, the contact area between screw surface and cortical bone increases with the increment of the peak stresses due to the shortening of the moment arm. Taken together, when placing miniscrews at a thinner cortical bone site, oblique insertion of the type B miniscrew presents the advantage of providing sufficient anchorage and primary stability. On the other hand, stability is likely to be achieved irrespective of the force direction when type A miniscrews are placed vertically.

In comparing the two different types of miniscrews, we found that the absolute values of the minimum principal stresses were reduced by 80–90% when type B miniscrews were placed obliquely and with upward traction as compared to type A miniscrews. This implies that, when type B miniscrews are placed, the induced stresses extended widely to the surrounding bone. This might be due to the unique thread shape of type B miniscrews. The screw thread of type B miniscrews is designed to set the proximal half angle to 35° and the distal half angle to 10°. Moreover, a previous study reported that when the proximal half angle was increased from 0° (vertically facing) to 30° (obliquely facing), the pulling strength of metal miniscrews increased by 16% and the pull-out force was evenly transduced to the peri-implant bone^46^. In addition, as compared to type A miniscrews with a conventional thread shape, type B miniscrews have a wider area in contact with the cortical bone when a pull-out force is exerted on the miniscrew. The proximal surface of the screw thread plays an important role in resistance against a pull-out force applied to the miniscrew. Therefore, primary stability may be enhanced by a unique thread shape in order to keep bone damage to a minimum, particularly when miniscrews are implanted obliquely.

Biomechanical models of living tissue are highly imperfect as they are based on a number of assumptions and simplifications. With respect to the present analysis, the following considerations should be described. First, the structures of the bone were modeled using isotropic material and a constant value of Young’s modulus over its whole thickness. In addition, there was no interface between cortical and trabecular bones. It was recently reported that the magnitude and modulus gradient of fascicular elastin in living tendon could contribute to the tensile mechanical response of the tendon, likely by regulating collagen engagement under load^47^. This means that a detailed representation of the material properties of the bone (anisotropy, the architecture of the cancellous bone, a discontinuous interface between the two bones) should be accounted for. Second, the contact between the miniscrew and the surrounding bone was established as a nonfrictional surface interface. However, in general, a certain amount of friction is present at the contact surfaces because the removal torque is greater than zero when the miniscrews are removed during and after orthodontic treatment^48^. Therefore, the results obtained in this study cannot be directly transferred to clinical practice without additional serious consideration informed by future rigorous research.

## Conclusion

Herein, we showed that type A miniscrews demonstrate the advantage of safety and secure stability when the screw is placed vertically, while type B miniscrews show the benefit of stress reduction when the screw is placed obliquely and with upward traction. Taken together, these data indicate that orthodontists should select and use the miniscrew most suitable for each patient in consideration of their bone properties.

## Data Availability

The data used for this study, though not available in a public repository, will be made available to other researchers upon reasonable request.
